# Geohash-Based Rapid Query Method of Regional Transactions in Blockchain for Internet of Vehicles

**DOI:** 10.3390/s22228885

**Published:** 2022-11-17

**Authors:** Chang Zhou, Huimei Lu, Yong Xiang, Jingbang Wu, Feng Wang

**Affiliations:** 1 School of Computer Science and Technology, Beijing Institute of Technology, Beijing 100081, China; 2 Department of Computer Science and Technology, Tsinghua University, Beijing 100084, China; 3 School of Computer and Information Engineering, Beijing Technology and Business University, Beijing 102488, China; 4 Department of Computer and Information Science, University of Mississippi, Oxford, MS 38677, USA

**Keywords:** query, blockchain, Geohash, Internet of Vehicles

## Abstract

Many researchers have introduced blockchain into the Internet of Vehicles (IoV) to support trading or other authentication applications between vehicles. However, the traditional blockchain cannot well support the query of transactions that occur in a specified area which is important for vehicle users since they are bound to the geolocations. Therefore, the querying efficiency of the geolocation attribute of transactions is vital for blockchain-based applications. Existing work does not well handle the geolocation of vehicles in the blockchain, and thus the querying efficiency is questionable. In this paper, we design a rapid query method of regional transactions in blockchain for IoV, including data structures and query algorithms. The main idea is to utilize the Geohash code to represent the area and serve as the key for transaction indexing and querying, and the geolocation is marked as one of the attributes of transactions in the blockchain. To further verify and evaluate the proposed design, on the basis of the implementation of Ethereum, which is a well-known blockchain, the results show that the proposed design achieves significantly better-querying speed than Ethereum.

## 1. Introduction

Recently, data authentication in the Internet of Vehicles (IoV) has attracted a lot of attention from researchers [1], e.g., malicious vehicles can seriously impact the usefulness of IoV information by sending fake locations [2,3,4,5,6]. To solve that, many works have introduced blockchain technology into IoV [7,8,9,10,11,12,13]. The blockchain is a distributed ledger technology that links the blocks storing transactions by hash, and supporting data integrity and traceability in the decentralization network environment [14]. A typical blockchain structure is Ethereum [15], which is open-sourced blockchain implementation. The main feature of Ethereum is that it uses the Merkle Patricia Tree (MPT) to build tree structures for storing account and trading information. The transaction information of the whole network is constructed into a chain structure, and any user can query all historical transactions that occurred.

In the scenario of IoV, vehicles are bonded to the geolocations, and they usually only focus on local information [10,16,17]. Compared with the information of the whole network, the transaction in the local area around the vehicle is more important to the user. However, the traditional blockchain structure cannot well support the query of transactions occurring in a specific area: users need to backtrack all transactions on the chain structure and then match the location where the transaction occurred. Moreover, the storage and access of geolocation data of transactions on the traditional blockchain need to be realized by external applications such as smart contracts [7,8,9,18,19,20,21], which further take up extra storage space.

Because of the high dynamics of the vehicles, the duration of the data link between vehicles is short, and the data querying efficiency of regional information is vital for the applications [22,23,24,25,26,27,28,29,30,31]. Therefore, how to improve the querying speed of information in a region is a key issue. There are some works that attempt to combine geolocation and blockchain in IoV by utilizing the out-chain structure such as the smart contract to maintain the geolocation data [7,9,18,32], however, they did not propose a satisfactory query method for regional information. We notice that in electronic maps, geographic regions are usually composed of map tile data of variable size [33,34]. On top of that, Our solution is to use geographic tiles as data indexes to improve the query speed of regional data. To achieve this goal, we utilize the Geohash code [35], which is a one-dimensional coding system and can efficiently represent a grid with arbitrary precision. In this case, the geolocation-related data stored in the chain is indexed by Geohash code, which can be easily queried by geolocations. Our previous study [33] also showed that the vector map data structure encoded by the Geohash code has good performance in terms of data storage and data compression. Moreover, To better evaluate the data structure and algorithm proposed in this paper, we modify the core structure of Ethereum to adopt the Geohash coding index.

In this paper, we propose a blockchain-based data structure for rapid querying of transactions that occurred in a specific region. Our main contributions are as follows:

1. Based on the Merkle-Patricia Trie (MPT) [15] structure, we design a region state trie (RST), which uses Geohash code to index geolocations of the hierarchical region as an attribute of trading.
2. On top of the proposed RST, we design a branch query method to efficiently query the geolocations of the hierarchical region. Furthermore, we design an account location trie (ALT) storing the location changes of each vehicle account, to support the querying of the historical trading location of each vehicle.

The rest of the paper is organized as follows. Related works are described in Section 2. Section 3 presents an overview of the geolocation blockchain structure, whose schematic design is then described in detail in Section 4. Section 5 discusses the experiment and evaluation. Finally, we conclude our paper in Section 6.

## 2. Related Works

Several works have been proposed for improving the efficiency of blockchain data queries [22,23,24,25]. In general, they can be divided into four categories. One is the off-chain query. The blockchain is only used to maintain data security, and an independent data query method is built off the blockchain, e.g., IBM [23] established four different databases for ledger index, status data, historical data, and block index to achieve separating query. Oracle [25] used CouchDB, which supports rich SQL queries, as the storage database to create indexes for both keys and values to improve query efficiency. The data of the blockchain is stored in the database of SAP [26] in the form of a virtual table to realize the off-chain query of the blockchain data. This method is mainly used for database suppliers. Although the query efficiency is improved, the data is transferred outside the blockchain, and its security depends on trusted entities very much. The second category is application query, which realizes different query functions by building different applications on the blockchain. Along this line, FlureeDB [27] is a graph database with blockchain core and ACID standard, which established a data snapshot related to the block or time point for each query to improve query efficiency. Swarm [28] is a blockchain system built to handle high-level files or layered collections of various types of metadata, using the form of the hierarchical set to improve the query efficiency. BigchainDB [24] added blockchain features based on big data distributed database. Nodes establish different indexes and query APIs according to query requirements to realize different application queries and improve query efficiency. The third category is contract query, which uses a smart contract of blockchain to optimize the query function. One example is vChain [22], which used the smart contract to simulate the underlying storage structure of blockchain and then established the inter-block index and intra-block index of simulated blocks. Blocks and indexes are reconstructed in the smart contract to achieve storage and query on-chain, but compared with being directly stored in the blockchain, the data amount reconstructed in the blockchain is much larger, and the internal query method of the blockchain is not improved. Different from vChain, G Gürsoy et al. [29] did not reconstruct the block structure, but only established an index structure for the stored content in the smart contract. The fourth category is sharding query, which divides the large-scale blockchain into multiple independent parts by sharding, to improve the query efficiency by reducing the amount of data accessed. For instance, chainSQL [30] divided the blockchain into a fixed number of shards, and improves data query efficiency by dispersing transactions into different shards. Monoxide [31] divided the user address space into multiple zones, and each zone established an independent blockchain, thereby reducing the amount of data in each zone. This method improves the query efficiency within the shard by reducing the amount of data in the shard, but its regional division does not consider the actual geolocation characteristics, and it is not suitable for the regional queries of geolocation-related information. In general, the methods mentioned above are dependent on credit entities, fail to improve the internal query method of the blockchain, and fail to consider the actual geographical location.

Several works have attempted to combine geolocation and blockchain in IoV through high-level structures such as smart contracts [7,9,18]. Table 1 is a comparison between our scheme and the technical schemes related to geolocation and blockchain. Those works have made contributions to improving the efficiency of blockchain data queries and combining geolocation and blockchain IoV applications. However, they did not well utilize geolocation and thus the region query efficiency is questionable.

In [7], the authors realized vehicle location sharing through the smart contracts, to improve the accuracy of cooperative positioning. Chukwuocha et al. [8] used the distance between the vehicle node and the event location to measure the message credibility, and divided multiple overlapping regions according to the road network to limit the credibility of cross-regional messages. Kudva et al. [9] read the geolocation and driving distance in the blockchain transaction simulated by the smart contract, and proposed the proof of driving consensus mechanism to improve the consensus efficiency, which shows that geolocation has great value in the application of IoV in the blockchain. Zhang et al. [36] implemented blockchain-based privacy location queries, designed location-based matching smart contracts, and returned matching results based on a horizontal and vertical grid coordinate checks. This is less efficient than the one-dimensional Geohash query method due to querying from two dimensions. These aforementioned studies store geolocations in the user data layer, and cannot use the blockchain’s security mechanism to ensure the security of location data, and thus there is no guarantee for the authenticity of the geolocation in blockchain transactions. Shrestha et al. [17] split the global blockchain into multiple independent blockchains according to location, where each independent blockchain receives messages only within its region, thereby improving message reliability and facilitating vehicle reliability verification. However, the article does not cover the work of querying regional information.

## 3. Structure Overview

**Figure 1:**
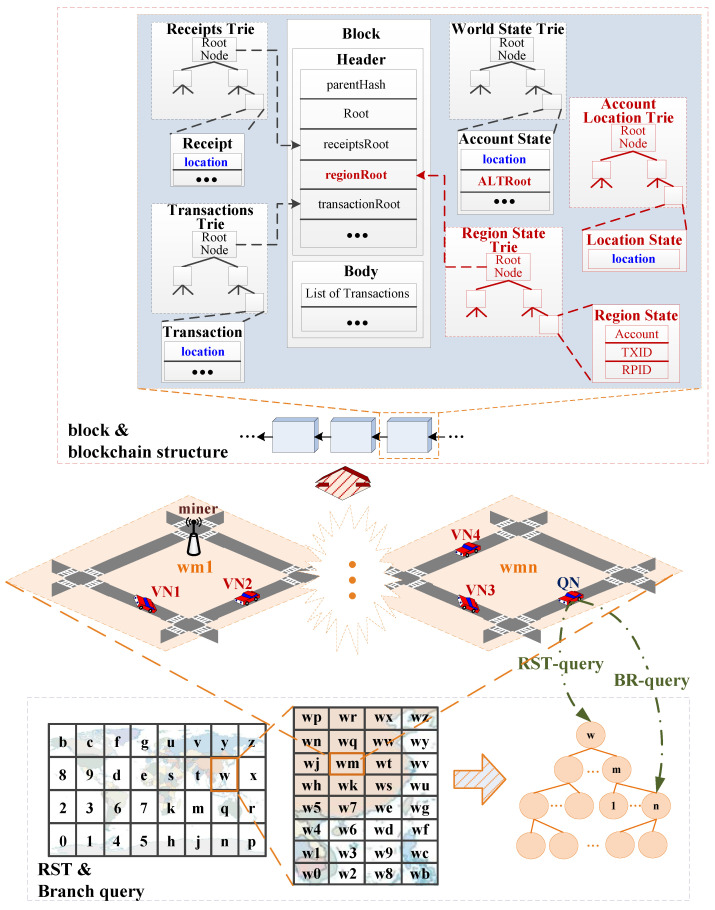
The proposed structural model. Where miner is used to packing the block, VN is a vehicle node, QN is a query node, RST-query means that the query node queries the regional information according to the RST method, BR-query means that the query node queries the regional information according to the branch query method.

As shown in Figure 1, the structure of Ethereum consists of three tries: receipts trie, world state trie, and transactions trie. On top of that, we add a region state trie (RST) to support the rapid querying of hierarchical geolocation-related (regional) data, and an account location trie (ALT) to support querying of the historical trading location of each vehicle.

Ethereum adopts the account-based data model. The block also contains the root hash of the receipt trie for the transaction execution log and the root hash of the state trie for the account state data, as shown in the block and blockchain structure in Figure 1.

As shown in Figure 1, we add the Geohash-encoded geolocation in the block attributes respectively: accounts, transactions, and receipts. We add the root hash of the index structure of the geolocation attribute (account location trie and region state trie) to the block to maintain the consistency and traceability of geolocations, which is convenient for geographic information querying and data credible verification. For other parts, including the consensus mechanism, our structure follows the existing settings of Ethereum.

1. Geolocation attributes are shown by blue location in Figure 1. Each vehicle is associated with blockchain accounts. We add the geolocation attributes named location to the account to meet the mobility of vehicles, where vehicle locations are recorded in chronological order to facilitate historical location queries. The geolocation is added in the transaction to achieve the purpose of adding geolocation attributes to the blockchain, which is also used to verify and update the account location of the transaction sender. The geolocation needs to be kept in the receipt as part of the transaction state information.
2. The root hash of the index structure of the geolocation attribute. Like the account balance, the account location trie reflects the change of account state. Therefore, its root hash does not need to be written into the block header, but to the account state. As shown by ALT Root in Header of Figure 1. The region state trie records the global changes within the geographic region, which has the same status as the global changes of account state. Its root hash needs to be written into the block header, as shown by regionRoot of Header in Figure 1.

To facilitate the querying of geolocation-related attributes, we build an index structure for them and design a branch query method according to the query requirements in different geographical regions.

If there is no index structure for the account location in the blockchain yet, vehicles need to go through two cyclic processes of traversing the block and traversing the transactions in the block when performing location verification or historical trading location querying. Therefore, an account location trie should be established for each account. Since vehicles in different regions pay more attention to the information of their geographical regions, the proposed RST store the geographical information according to the query requirements of geographical regions. And we further design a branch region information query method to reduce the overhead and improve the efficiency of branch region information querying.

In the proposed ALT, a MPT structure with a time as key and geolocation as value is established for each account to realize the function of supporting geographic region information query within the blockchain (See details in Section 4.1). In the proposed RST, a MPT structure of a region state with Geohash code features and hierarchical relationship of geographic regions, which is represented by the amount of transactions within the specified geographic region. We use the Geohash encoding of the geolocation as key, and the region elements list in the current region (including account list (Account), transaction list (TXID), and receipt list (RPID)) as the value in this structure (See Section 4.2 for details).

The branch query method we designed requires that the branch index structure of the region state trie corresponding to the geographic region be saved according to the geolocation of the node, to narrow the query scope and improve the query efficiency (See Section 4.2.4 for details).

## 4. Design of the Proposed Structure

### 4.1. Account Location Trie (ALT)

To support the querying of the historical trading location of each vehicle, the trading location is treated as the attribute of the corresponding account in the blockchain. Like the balance of the account, all account locations are also written into the independent state database of each account as state information.

**Figure 2:**
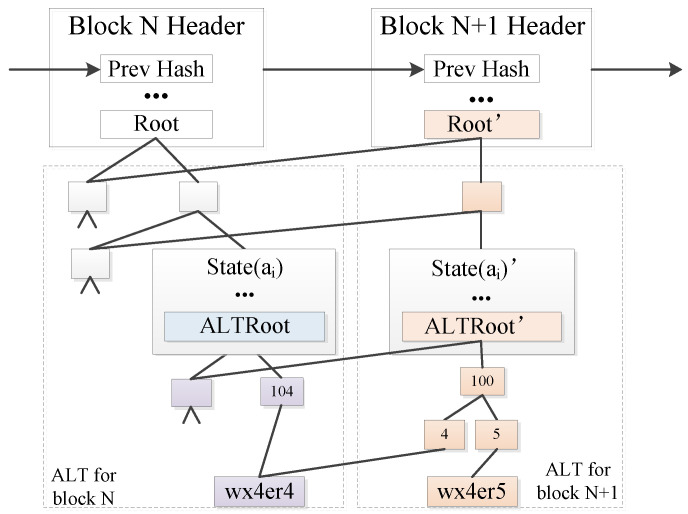
Schematic Diagram of ALT Storage Changing.

1. Structure of ALT.
  As the core data structure of Ethereum, MPT is a tree structure of single value identification key/value pairs formed by the combination of Merkle Tree and Patricia Tree. The advantages of the MPT structure are the query efficiency brought by the compressed prefix and the fast location of differences brought by hash merging. Therefore, we use the MPT structure to build ALT to achieve fast query of geographic information.
  When a transaction happened, ALT takes the record time of the account geolocation as the index, where the account geolocation of the transaction sender verified by the miner node is stored in the leaf node of the trie. It then combines the hash values of two adjacent leaves into a string and stores the hash value of the string in the parent node of the trie. The adjacent parent node repeats the above hash calculation process until the root hash value is obtained. To this end, the change of the entire ALT can be identified by the root node.
  As shown in Figure 2, the storage of ALT consists of on-chain storage and index structure storage. The on-chain storage part is the hash value Root of the root node of the state trie in the block header. The dashed box is the index structure, and the node of the blockchain will maintain the latest index structure of ALT locally. The change of the account location will cause the change of the hash value of the root node of ALT in the account state ALTRoot, which will lead to the change of the hash value Root in the block header. It’s still shown in Figure 2, the blockchain adds a new block numbered N+1. Since a new location record with a new time of 105 and a location of wx4er5 is added to ALT, the hash value of the root node of ALT changes from ALTRoot to $ALTRoot^{\prime }$, and the hash value of state tree root node changes from Root to $Root^{\prime }$.
2. Querying of the account location.
  The account location query can be divided into two types: the latest location query and the historical location query. Since the latest location of the account is an attribute of the account, it can be obtained directly. The historical location needs to be queried based on the specific time. First, it queries the latest account state, reads the root node of ALT from the account state, then queries ALT with the specified time as the index, and finally returns the index.

### 4.2. Region State Trie (RST)

The purpose of the ALT is to implement a location query related to the specified account. It can also be used to query the region state without an independent query function for the region state. However, it is not efficient for querying regional information due to the querying process based on two dimensions of account and geographic region. We design the region state trie (RST), which uses the Geohash encoding as the index of MPT, to support the rapid querying of the hierarchical geolocations (region) state. RST records the region state information within a certain geographic region, optimizes the MPT indexing method, and makes it efficiently support the prefix query, the latest regional information query and the branch query in the core of the blockchain. Furthermore, we reduce the impact of external operations on the security of the blockchain by providing an interface for external access to facilitate application calls.

#### 4.2.1. Structural Design

**Figure 3:**
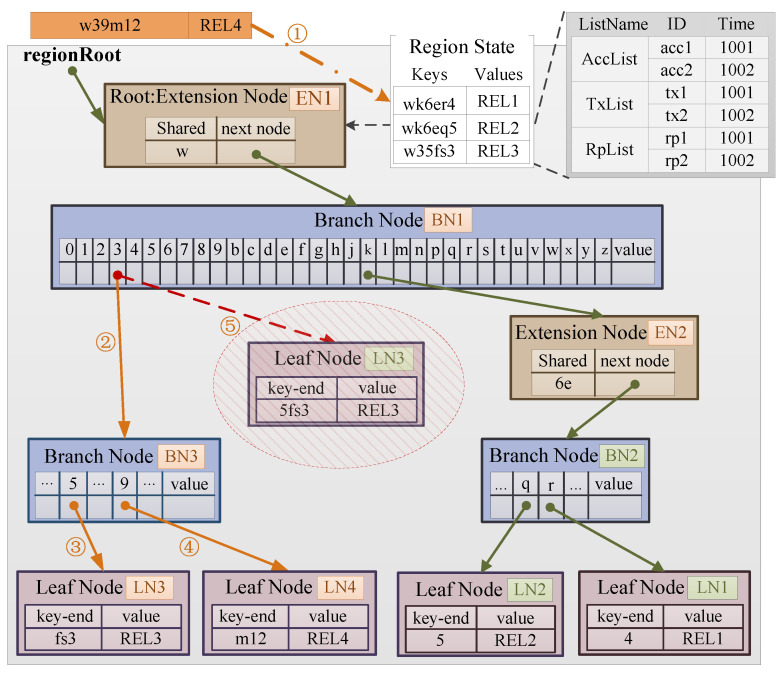
Schematic diagram of the region state trie structure.

Figure 3 shows a schematic diagram of the RST structure. In general, we use the Geohash code to index the geographic region, branch node of RST and the geolocation attribute of each block. We further design a region elements list (REL) by using the bucket of MBT (Merkle Bucket Tree) [23] to store multiple region state information in the same leaf node of the trie. RELs are arranged in chronological order, which is convenient to query the latest information. We design a prefix query method to query the information of multiple leaf regions in the RST. We design the branch query method to improve query efficiency of the region state in the specified branch region.

The key in RST is encoded with a 14-byte Geohash since the 14-byte Geohash code can meet the centimeter-level accuracy requirements for geolocations at all latitudes on the Earth [33]. We adopt the recursive length prefix [15] storage method in RST to facilitate the storage requirements of different data amount.

The RST takes the REL of the state information in the region as value. There are three types of nodes in RST, as detailed below.

**Extension Node**: Record the common prefix (shared) and followed branch node index (next node) of all unwritten nodes when traversing from the root node to this node. As part of the key, the common prefix also uses Geohash code. The purpose of constructing extension node is to expand the branches of the tree structure, and thus each extension node will follow a branch node.

**Branch Node**: Record the branch path traversed by different keys to this node. According to the Geohash encoding rules, the first 32 items of this node record the branch, and the 33rd item records the key end flag. It is worth noting that if there is no combined common prefix before the branch junction, there is no extended node before the branch node. As shown in the branch node BN3 in Figure 3, the previous node is the branch node BN1.

**Leaf Node**: Record the remaining key encoding value with no branch and the corresponding REL. An RST with only one item is a leaf node. When the RST has only one item, it is a leaf node.

#### 4.2.2. Construction and Update of RST

As we mentioned before, the region state information is stored in the RST in the form of REL. REL consists of the account list, transaction list and receipt list. The region element contains two attributes: ID and time. ID refers to the account ID, transaction ID and receipt ID in the account list, transaction list and receipt list respectively. REL contains all region elements in the geographic region, and there is no duplicate data in any two RELs. For the account list in the REL, only the latest transaction time of the account is retained in the same account list to indicate the activity of the account.

**Construction of RST**. The construction of RST includes two parts of top-down storage path planning and bottom-up node value hash merging.

1. Storage path planning. The storage path planning refers to the construction process of all branch nodes starting from the root node to the final leaf node of the RST in accordance with the geolocation encoding REL. The initial state of the RST is empty, and the first data is written to the root node. When there is an REL in the region to be planned, this REL is updated; otherwise, the geographic region encoding where the current blockchain is located is used as the common prefix to plan the storage path. The storage path planning process of the RST is summarized in Algorithm 1.
2. The node hash value merging process of the RST is consistent with that of ALT. Both of them need to start from the leaf node and combine the adjacent nodes to calculate the merging hash and pass it up until the root hash value is obtained.

**Update of RST**. The RST update process includes three parts: storage path update, update of REL and update of the node hash value. When the storage path of the RST generated by the key to be updated does not overlap with the existing path in the tree, a new branch of the RST needs to be established, which is the storage path update. When the storage path of the RST generated by the key to be updated is the same as the existing path in the tree, the new REL needs to be updated to the original REL, which is the REL update. The above two update processes need to be accompanied by the update process of node hash value.

Take the item to be updated (step ➀) where key is w39m12 and value is REL4 in Figure 3 as an example to illustrate the update process of the RST. When querying the branch 3 of branch node BN1, the record is not empty, one needs to compare the key-end of the leaf node. Since the key encoding of REL4 is read to 9m12, the key of the original leaf node LN3 is 5fs3, and the first encoding is different, it will enter the storage path update process, that is, a branch node is established (step ➁), and then leaf nodes LN3 and LN4 with keys of fs3 and m12 are created respectively (steps ➂ and ➃). Then, it enters the node value hash update process, that is, the hash values of leaf nodes LN3 and LN4 are written into the 5 and 9 branches of branch node BN3 respectively. Finally, the original leaf node LN3 is deleted (step ➄), and the update process is done.
**Algorithm 1** Storage path planning algorithm of RST**Require:** R, w  1:**function**Storage_path_Planning(R, *w*)  2:    //*w* is the Geohash region encoding that *R* belongs to  3:    //cur_ Node is the node with the longest prefix of *w*  4:    //sub_region is *w* removing the prefix of cur_node and *w*  5:    **if** sub_region.len>0 **then**  6:        LeafNode[sub_region(1:end)]=R  7:        BranchNode[sub_region(0)]=                  LeafNode[cur_node.key(1:end)]  8:        **if** cur_node.type≠BranchNode **then**  9:           LeafNode[cur_node.key(1:end)]=cur_node 10:          BranchNode[cur_node.key(0)]=                  LeafNode[cur_node.key(1:end)] 11:        **end if** 12:    **else** 13:        **if** cur_node.type==BranchNode **then** 14:           BranchNode.value=R 15:        **else** 16:           R is stored into REL 17:        **end if** 18:    **end if** 19:**end function**

#### 4.2.3. Prefix Query

**Figure 4:**
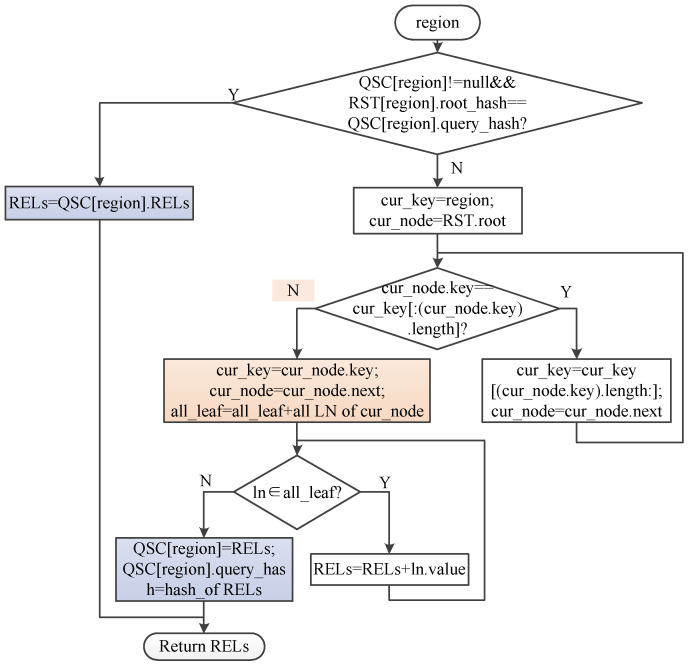
Flow chart for prefix query of RST.

The RST supports MPT query and prefix query. MPT query requires the same length of encoding bytes of the query region and the number of encoding bytes of the key of the construction tree. And the query result is empty or the value of the leaf node. To query different levels of regional information according to the prefix of the geolocation encoding, we design a prefix query method. Prefix query requires the Geohash encoding of the region to be queried as the prefix of the key of RST. And the query result is empty or the combined value of all leaf node values of the branch where the prefix is located, as shown in the pink part in Figure 4. Prefix query includes two parts: RST query and query state cache.

1. RST query. A prefix matching method is designed to implement the prefix region query of the RST. We use input to represent the encoding of the region to be queried, and cur_key to represent the encoding of the current key to be queried in the prefix matching process. The entire prefix matching process is a byte by byte comparison process from the first byte of the input encoding and then getting the cur_key. When comparing with the root node of RST for the first time, input is the cur_key. When the key of parent node is the prefix of cur_key, cur_key is the remainder of the parent node without prefix. If the leaf node is not reached after traversing input, we take the key of the child node of parent node as cur_key, and traverse the child nodes one by one until all the leaf nodes of parent node are traversed.
2. Query state cache. Obtaining the regional geographic information of the IoV ultimately requires querying the REL. When the REL to be queried has not changed, caching the query state can improve the query efficiency of the REL. We design a query state cache method to temporarily save the query results of the REL. As shown in the blue part in Figure 4, we use QSC to represent the query state cache list, region is used as the key of region state cache to record the region encoding of the query request, RELs stores the REL corresponding to the region. query_hash is the hash value of the query state cache. When the region information is queried, we check whether the element exists in the query state cache list firstly. If not empty, the hash value of the query state cache is compared with the node of RST. If the hash value of the corresponding element of the RST is consistent with the hash value of the corresponding element in the query state cache list, the query state cache result is returned directly. If the element does not exist in the query state cache list or the element exists but is not the latest state, the query process of the RST needs to be completed, and then the hash value of the query state cache list and the query state cache in the cache should be updated.

#### 4.2.4. Branch Query

Compared with geographic information outside the region, vehicles need geographic information of their own regions provided by blockchain nodes to meet the requirements of IoV services. We propose the branch query method, where the blockchain node caches the region state data of their own geographic region so as to improve the efficiency of branch query.

**Figure 5:**
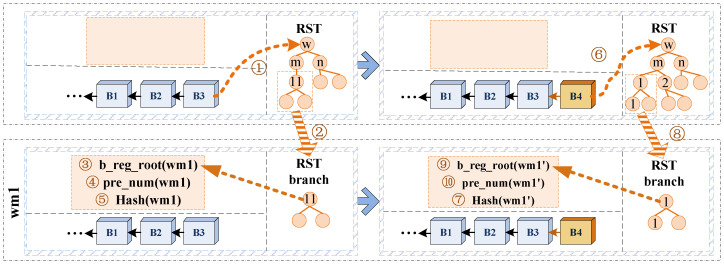
Schematic diagram of branch query method.

1. Branch region cache. The contents of the branch region cache include the root node, the RST branch of the geographic region where the node is located, and the branch node prefix. In this paper, we define the branch node prefix as the encoding length of all parent node keys of the RST branch which is cached by the node in the global RST.
  Figure 5 shows a schematic diagram of the establishment and update of the branch query method. In the figure, the node whose geographic region encoding regionID is empty adopts the global RST query method, and the node whose regionID is wm1 adopts the branch query method. A blue dashed box in the figure is the storage state of the blockchain node at some point, including two parts: the orange box represents the state cache of the branch region of the current node, and the lower part represents the chain link relationship of the blockchain. The process of establishing branch region cache for node wm1 in Figure 5 is as follows: 1 read the root node of the global RST from the latest block B3; 2 query the branch of wm1 in the global RST; 3 obtain the root node of current region, namely b_reg_root(wm1) with key 11; 4 get the prefix-encoded bytes of the branch region node pre_num(wm1); and 5 calculate the hash value Hash(wm1) of the root node of the branch of RST.
  After the node using the branch query method exits, its branch region cache is invalid, and the cache needs to be re-established when starting again. The algorithm for finding the root node of the branch region state and the prefix of the branch node is shown in Algorithm 2.

**Algorithm 2** Branch Region State Cache
**Input:** RST.root, regionID**Output:** b_reg_root, pre_num
1:v_reg = regionID2:pre_num=03:b_reg_root = RST.root4:**while**v_Reg is not the prefix of b_reg_root.key **do**5:    pre_num = pre_num + len(b_reg_root.key)6:    v_reg = v_reg remove the prefix of b_reg_root.key7:    b_reg_root = b_reg_root.next8:
**end while**
9:**return** b_reg_root, pre_num

2. Branch region update. When the region state branch corresponding to regionID of the node using the branch query method changes, the local branch region state cache needs to be updated. As the structure of the RST will be changed, the hash value and prefix of the branch corresponding to regionID will be changed, too. Therefore, both the hash value of the branch and the prefix of branch node need to be updated. As shown in Figure 5, after the node wm1 completes the synchronization process of the block B4, a new RST is generated (step ➅), and then the root node hash of the branch of RST $Hash(wm1^{\prime })$ needs to be calculated (step ➆). $Hash\left(wm1^{\prime }\right)\neq Hash\left(wm1\right)$ indicates that the branch has changed, re-search the branch of RST of the node (step ➇) and update the branch region state cache to $b\_reg\_root(wm1^{\prime })$ and $pre\_num(wm1^{\prime })$ (step ➈ and ➉, respectively).
3. Branch query. If the Geohash encoding of the branch region and the Geohash encoding prefix of the region to be queried are the same, the fast query of the state of the branch region can be achieved by using the locally cached RST branch of the region. It is not necessary to start the query from the root node of the RST, but only from the branch node of the cache. As shown in Figure 5, the node of wm1 queries the region state of the range of wm1 or wm11, which is the branch query. When the node does not set the region to be queried, or when the actual query region is the upper region of the node’s region or other regions that are not prefixed by the node’s current branch region, (because there is no corresponding region state cache), it needs to be queried in the global RST of the corresponding region state.

### 4.3. Discussion on Data Security

We analyze the storage security and query security of the geolocation blockchain in this section.

**Storage security.**

1. The blockchain adopts asymmetric encryption algorithm, which can resist many traditional security attacks, and its distributed storage structure ensures storage security [7,8,9,10,11,12].
2. Locations are written into blocks through transactions, which can achieve consistency and tamper-resistance according to the distributed consensus mechanism and synchronization mechanism.
3. The geolocation attribute is added in the core of the blockchain. Although there is a risk of exposing the location, the authenticity of the transaction is increased by adding the location attribute to the transaction, which provides security guarantee on the other hand.

**Query security.**

1. Privacy. The original blockchain does not provide location information and there is no risk of exposing the location. We add location attributes inside the geolocation blockchain. Although the authenticity of the transaction can be proved by the location, if the location access is unrestricted, there is a risk of privacy leakage. Therefore, according to the application scenarios of location information, we divide location access rights into internal index usage and external usage depending on whether it is one of the two parties in the transaction. During the internal indexing of the blockchain, the access to location information is unlimited. In other cases, only the both parties can access the specific location information, so as to achieve the purpose of restricting the use of location information.
2. The input is non-repeatable. This is because only one transaction and one input of location can occur in the same account at one time, especially ALT takes time as the key, and the key is not repeated. That is, there will be no case that one time corresponds to two locations in ALT.
3. Query reliability. When querying the state of the same region, any two nodes return the same query results. Assume that there are two nodes that query the same region state information with different results. Since the honest nodes in the whole network account for the majority, the root node hash of the RST of all nodes are the same, so there will be no different branches. This contradicts with the assumption, which also proves the query reliability.

It can be seen through the above security analysis that our solution has no impact on the security of the blockchain, and the reliability of the query can also be guaranteed. Although there is a risk of exposing account privacy, it can be improved by adding simple query restrictions.

### 4.4. Discussion on Query Time

We analyze and discuss the query time of querying region state information in the no-location index method, ALT method, the global RST method, and the branch query method in this subsection.

1. No-location index method.
  Existing blockchains such as Ethereum have no indexing method for geolocation, which we refer to as a no-location index. There are two ways to add location information to Ethereum. One is to use smart contracts to store locations, but they cannot be associated with ordinary transactions. If smart contracts are used to achieve storage and access data on-chain, the amount of data storage and storage cost will be increased. The other way is to store location information in the transaction in the form of extra data. The location data needs to be stored in the recursive length prefix encoding format and then written into ordinary transaction. The query process mainly includes a cyclic search process of block query and transaction query. When obtaining the transaction location, the recursive length prefix decoding process needs to be completed, and then compared with the range to be queried. We select the latter one for comparative analysis.
  Assuming the number of blocks is *m*, the transaction amount is *n*, the average transaction amount packaged in each block is $\frac{n}{m}$, then the query time complexity is $O\left(m\times \frac{n}{m}\right)=O\left(n\right)$.
2. ALT method.
  The steps to query the region state with the help of ALT are as follows: First, get all account IDs. To get the state of the entire region from the perspective of ALT (mainly refers to the number of transactions), we first need to count all historical account information in the region. Second, get all transactions of the account. We need to get the start and end time of the query. The start time is when the account sends the first transaction, and the end time is when the account sends the latest transaction. Due to the randomness of the transaction, we can determine it from the block generation time. We choose block 0, i.e., the timestamp of the genesis block as the start time, because genesis block does not contain any transactions. We choose the timestamp of the latest block in the current blockchain as the end time to achieve full coverage in time. Third, transaction location filtering. In the process of step 2, it is also necessary to filter transactions according to the query region. According to Geohash encoding rules, it is only necessary to check whether the query region is the prefix of the transaction location.
  Assuming the number of blocks is *m*, the transaction amount is *n*, the number of accounts is *a*, then the average transaction amount of each account is $\frac{n}{a}$. ALT is a Merkle tree, which has the query time complexity of O(logN), with *N* being the number of leaf nodes [37]. ALT takes time as the key, and each account has an independent ALT. Therefore, the number of leaf nodes is the transaction quantity of the account, so the query time complexity of each account is $\log\frac{n}{a}$, and the overall query time complexity is $O\left(a\log\frac{n}{a}\right)\approx O\left(\log n\right)$. Thus, the query time complexity of ALT method is less than that of the no-location index method.
3. Global RST method.
  Since there is no loop process in the process of storage path planning process of the RST which is summarized in Algorithm 1, its time complexity is O(1). The planning process is independent of the query process and will not affect the query time complexity of the Global RST method.
  The RST is also a Merkle tree, and the Merkle tree has the query time complexity of O(logN), where *N* is the number of leaf nodes. Since the RST is stored in bucket structure at the leaf node, we suppose the number of transactions stored in the REL at the leaf node in the RST is *S*. Then the number of leaf nodes in the RST is N/S, and the query time complexity of the global RST is O(log(N/S)). Therefore the query time complexity of the global RST method is less than that of ALT method.
4. Branch query method.
  The time complexity of the algorithm for finding the root node of the branch region state and the prefix of the branch node which is summarized in Algorithm 2 depends on the encoding length of the branch region to be queried and the length of the common prefix in RST. In the worst case, if the coding length of each branch in RST is 1, the time complexity of Algorithm 2 is O(n). Since this process is an action that can be completed when the node is started, it does not need to wait until the region information is queried, and it will not increase the query time of the region information.
  Supposing the number of leaf nodes of the Global RST method is N/S, and there are at most *L* branches in each layer of RST. According to Algorithm 2, we assume that the length of the prefix region code cached in the branch region query method is *P*. Then the number of leaf nodes of the RST cached in the branch region query method is $\frac{1}{L^{p}}*\frac{N}{S}$, which is less than that of the Global RST method. We can conclude that the query time complexity of the branch query method is $O\left(\log_{}\left(\frac{1}{L^{p}}*\frac{N}{S}\right)\right)$, which is less than that of the Global RST method.

In the case of the region state query with the same transaction content and the same amount of data, the order of query time is no-location index method > ALT method > global RST method > branch query method. Compared with the query time complexity of the Merkle tree in literature [37], our query method is in the same order of magnitude as literature [37], which shows that our query method has no impact on the query advantages of the original Merkle tree although it adds location information.

## 5. Experiments and Evaluation

We have implemented the proposed data structure based on the Ethereum. In this section, we first introduce the experimental environment and experimental data used in this work. Then, we evaluate the performance of the geolocation blockchain structure with the region state query function.

### 5.1. Experimental Setup

1. Experimental environment. Our prototype framework is implemented on the basis of Ethereum version 1.9.12 (16 March 2020) which is written in Go language. The physical environment of the experiment is a desktop computer with 4-core Intel Core i5-4690k 3.5 GHz processor and 7.7 GB memory, running on Ubuntu 14.04.
2. Data generation. It is set that the vehicle sends a position transaction every 200 ms, and the speed limit of the general urban road is 30–50 km/h [7], so we set the average speed of 40 km/h as the vehicle speed. The vehicle trajectory is the position sequence represented by the 14-byte Geohash encoding continuously generated in the specified region according to the specified speed. The vehicle node writes the vehicle location into the transaction to record the movement of the vehicle. The no-location index blockchain stores location data into the blockchain in the form of additional data of ordinary transaction, and the geolocation blockchain structure stores it in the form of location attributes of the transaction. Each vehicle node sends a transaction to the blockchain every 200 ms.
3. Data acquisition. To evaluate the performance of the prototype framework, we mainly use three metrics: (1) Taking the time when the same content and the same number of transactions are written into the blockchain as the build time to compare the time cost when building different blockchains. (2) Taking the amount of on-chain data and local-data which are written into the blockchain with the same content and the same number of transactions as the data amount to compare the space cost when building different blockchains. Since the data content stored by different blockchain nodes is different, we make statistics from the data amount on-chain and the local data amount, respectively. Data amount on-chain: only the data amount of the block itself is counted. Local data amount: count the complete data amount saved locally, including state data. (3) Taking the query time of the amount of transactions in the same region as the query time to express the query efficiency of different types of query methods on regional information. For the statistics of the build time and the data amount, we calculate the situation separately when the amount of transactions is 8000, 16,000, 24,000, 32,000, 40,000. For the statistics of query time, we set the query node to complete the regional information query every 10 s, and take the average value for 200 consecutive queries.
4. Experimental scenario. Four vehicle nodes represent vehicles in four different regions, which generate vehicle location in their respective regions (the movement range of each node’s location is four 5-byte Geohash encoding regions, with a region of about 80 ${\mathrm{km}}^{2}$), and send a transaction every 200 ms. One fixed node acts as a miner and is responsible for packaging transactions. One query node is responsible for the query work.
5. Comparison protocol. As in the previous section, our comparison protocol includes the original no index method, ALT method, global RST index method and branch region query method.
  Original no index method (ORG) is as discussed in the first paragraph of Section 4.4. In the original Ethereum, the location data of the transaction is stored in the form of additional information. Since there is no index for additional information in the blockchain, the query needs to be completed with blocks and intra-block transactions. This method with no dedicated location query is referred to as the original no index method.
  ALT index method (ALT) is as discussed in second paragraph of Section 4.4. In the geolocation blockchain, ALT is used to query the region state from the perspective of the account. This method is called ALT index method.
  Global RST index method (RST) is as discussed in Section 4.2.1, Section 4.2.2, Section 4.2.3. In the geolocation blockchain structure with RST, the nodes use a global query method to complete the region state query work, which is called the global RST index method.
  Branch region query method (BR) is as discussed in Section 4.2.4. In the geolocation blockchain structure with branch query method, the nodes use the branch query method to complete the query work for the cache of the current branch region state.

### 5.2. Experimental Results

In this section, we evaluate the performance of the geolocation blockchain structure prototype supporting region state query from different aspects. First, we measure the two performance indicators of build time and data amount of the no-location index blockchain and the geolocation blockchain. Then we perform a quantitative analysis of the query time of the original no index method (ORG), ALT index method (ALT), global RST index method (RST) and branch region query method (BR) when querying different geographic regions under different transaction amount. Finally, we further analyze the impact of node region range and query range on branch region query method.

**Figure 6:**
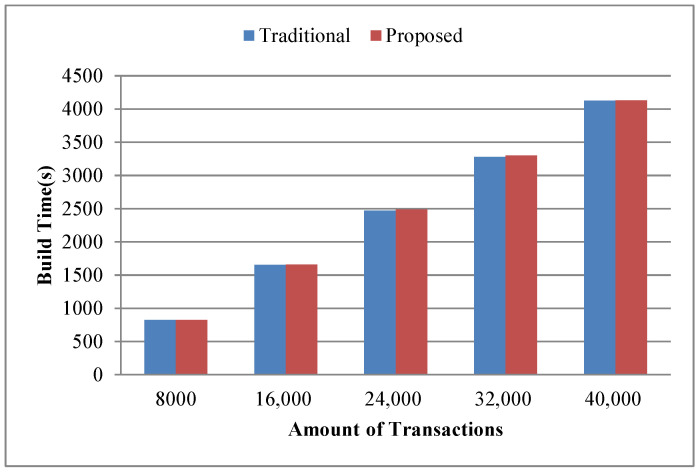
Build time with different amount of transactions.

**Figure 7:**
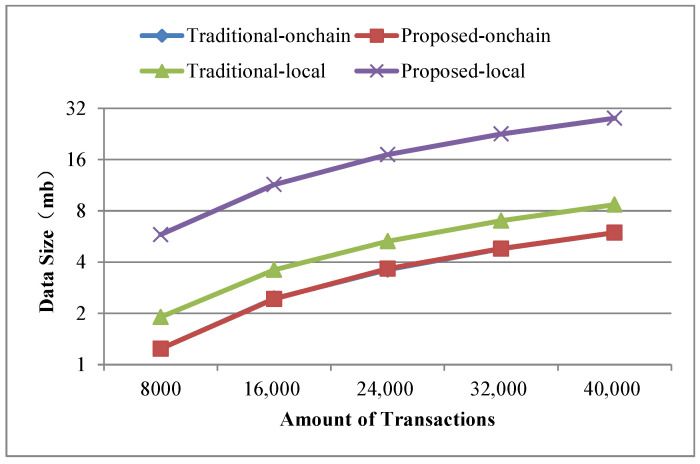
Data amount with different amount of transactions.

**Figure 8:**
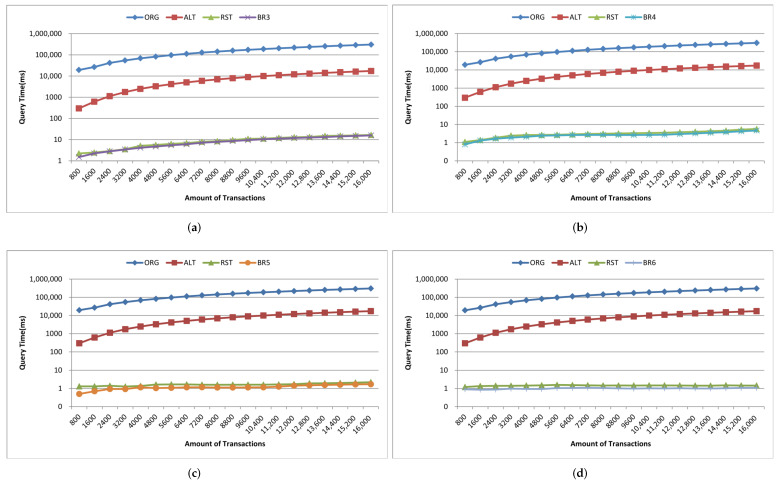
Query Time with Different Amount of Transactions in Various Geographic Regions. (**a**) The query range is geographic region of the 3-byte Geohash encoding. (**b**) The query range is geographic region of the 4-byte Geohash encoding. (**c**) The query range is geographic region of the 5-byte Geohash encoding. (**d**) The query range is geographic region of the 6-byte Geohash encoding.

**Figure 9:**
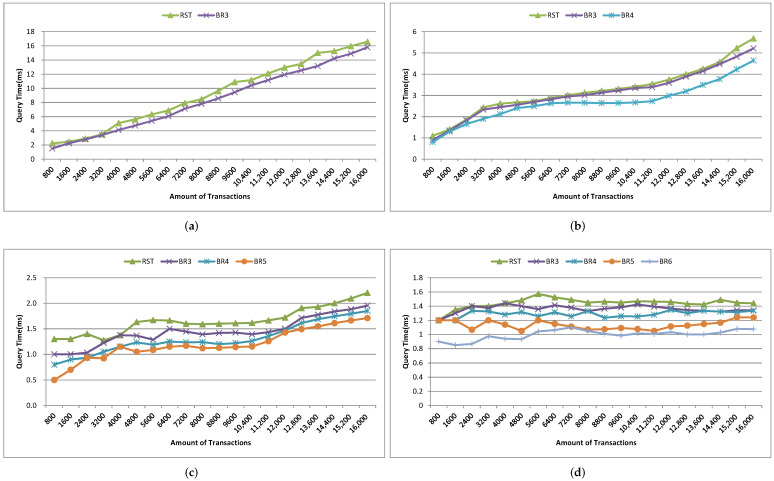
Query time of the amount of branch region query method with different amount of transactions in specified geographic region. (**a**) The query range is geographic region of the 3-byte Geohash encoding. (**b**) The query range is geographic region of the 4-byte Geohash encoding. (**c**) The query range is geographic region of the 5-byte Geohash encoding. (**d**) The query range is geographic region of the 6-byte Geohash encoding.

1. Build time. We take the successful packaging time of the same number and the same content of transactions as the build time, and make statistics on the build time of five cases, such as 8000, 16,000, 24,000, 32,000, 40,000 transactions separately. Figure 6 shows the comparison of build time. The build time and time growth trend of the geolocation blockchain structure and the no-location index blockchain are basically the same, which both increase with the increase of the transaction amount on the whole. The build time of the geolocation blockchain structure increases about 0.31% on average compared with the no-location index blockchain, indicating that although the index structure is added to the geolocation blockchain structure, the build time does not increase significantly.
  Since our research did not modify the consensus mechanism, one miner and multiple miners had little impact on the test of construction time when the sending transaction speed was consistent, so we did not involve the multi miner experiment in this article.
2. Data amount. The experimental settings of data amount statistics are the same as that of building time statistics. The comparison results of data amount are shown in Figure 7.
  In terms of the data amount on-chain, as shown in Figure 7, the amount of on-chain data increases as the number of transactions increases in both cases. Compared with the no-location index blockchain, the on-chain data amount of the geolocation blockchain structure is slightly increased, with an average increase of about 0.27%. This is because the geolocation blockchain structure only adds the root nodes of ALT and RST in the block header, and the amount of data contained in the transactions included in the block body keeps the same. Therefore, the geolocation blockchain structure has no significant impact on the data on-chain.
  From the perspective of local data amount, roughly speaking, the data amount of the two methods increases with the increase of the number of transactions. The data amount of geolocation blockchain structure has increased significantly, which is about 3.2 times more than that of the no-location index blockchain on average, indicating that the data amount of database of the geolocation blockchain structure is significantly higher than that of the no-location index blockchain. The reasons are as follows: (1) the storage of ALT built for each account will continue to expand as the number of account transactions increases; and (2) the storage of RST will continue to expand according to the increase of the number of transactions in the region. The statistical results of the database data amount show that the index structure of the geolocation blockchain structure trades off space for time.
3. Query time. Query time refers to the time spent by different index methods to query the amount of transactions within the same geographic region when the amount of transactions on-chain is the same. Here we count the cumulative average value according to the fixed interval amount of transactions and count the query time of the transaction quantity in the geographic region with the query range of 3–6 byte Geohash encoding respectively. The statistical results are shown in Figure 8. The branch region selected by the region node of the branch query index in Figure 8 is the same as the Geohash range selected by the query region. And, in the four query result diagrams in Figure 8a–d, the query range of nodes corresponding to the branch region index is also different.
  Overall, when the query range is a 3-byte Geohash range, the amount of transactions contained in the region grows fast due to the large size of the region. Therefore, the query time of the four query methods increases greatly with the increase of the amount of transactions in this range. When the query range is a 6-byte Geohash range, the query time increases, but at a small rate, because the region is small and the amount of transactions contained within the region grows slowly.
  Comparing ALT index and original no index methods, when the query region is in the range of 3–6-byte Geohash, the average query time of original no index is 19 s–306 s, while the average query time of ALT index is 0.3 s–17 s. The average query time of ALT index is about 5.3% of original no index, which shows that ALT index has greatly improved the query efficiency compared with original no index on querying region state.
  Comparing global RST index and ALT index, when the query region is in the range of 3–6-byte Geohash, the query time of global RST index is 1.4 ms–9.2 ms on average, which is at least 99.8% less than that of ALT index, indicating that the global RST index of RST has better region query efficiency.
  Comparing branch region index and global RST index, when the query region is in the range of 3–6-byte Geohash and the query range of region index is also in the corresponding range of 3–6-byte Geohash, the average query time of branch region index is 1 ms–8.4 ms, which is about 21.7% less than that of global RST index, showing that the branch region index also improves the efficiency of RST.
  These experimental results have validated the discussion on query time in Section 4.4.
4. Analysis of branch region query method.
  We further evaluate the branch region index query method under the geographic region of different region nodes in the same query range. The statistical results are shown in Figure 9. The BR3–BR6 in Figure 9 denote the length of Geohash encoding used to represent different regions during the region state information query process.
  When the amount of transactions is small (about 1200), the region query time of global RST index is the same as that of branch region index when the range is 3-byte Geohash encoding. With the increase of the amount of transactions, the query advantages of the branch region query method are gradually improved. It can also be seen from the results that the query time is greatly affected by the amount of transactions within the query range. When the amount of transactions within the query range changes little, the query time is almost the same. For example, when the amount of transactions in Figure 9b is 5600–10,400, the query time of BR4 is basically the same. Also, in Figure 9d, because of the small query range, the amount of transactions in the region is small and changes little, making the query time not change much.
  When the region of node is consistent with the query range, the query time of branch region query method is reduced by about 21.7% compared with that of the global RST index on average. When the query range is in the 4–6-byte Geohash encoding range and the range of region index is three byte, the query time of branch region query method is about 7.6% less than that of the global RST index on average. When the query region is in the range of 5–6-byte Geohash encoding and the range of branch region query method is four byte, the query time of region query method is about 16.2% less than that of the global RST index on average. When the query region is in the 6-byte Geohash encoding range and the range of branch region query method is in the 5-byte, the query time of region query method is reduced by about 21% on average compared with that of the global RST index. It shows that when the range of region query method is consistent, the branch region query method has higher query efficiency than global RST index and when the query range is about the same as the region range, the branch region query method efficiency is higher. This plays a guiding role in region state query and the region selection of the region node.

### 5.3. Discussion

The experimental results above show that in terms of construction time and the amount of data on-chain, the geolocation blockchain only has marginal growth of 0.31% and 0.27% on average compared with the no-location index blockchain. In terms of the local data amount, due to the additional index structure of the geolocation, the data amount of the geolocation blockchain is 3.2 times that of the no-location index blockchain on average, indicating that the geolocation blockchain uses storage space in exchange for query efficiency. For query time, the query efficiency of the ALT index of the geolocation blockchain is 5.3% of the original no index query. Compared with the ALT index, the query time of the global RST index is reduced by 99.8%. Compared with the global RST index, the query time of the branch region index is reduced by 21.7%, indicating that the geolocation blockchain has observable advantages in region state query. In addition, we have further concluded that the more consistent the geolocation range of the node is with the branch query range, the better the query efficiency is. The effectiveness of geographical location blockchain in regional information query is validated by the discussion of data security, query time, and experimental results.

## 6. Conclusions

In this paper, in order to quickly obtain region state information in IoV, we design a region state trie based on the Geohash coding system to index the hierarchical geolocation attributes in blocks. We further design an account location trie to support the querying of historical trading locations. Furthermore, we proposed a branch query method to improve the efficiency of region query. Our design is implemented on the basis of the open-sourced Ethereum, instead of using logical structures such as establishing smart contracts on the upper layer, which is more convenient for future development and application.

At present, the query of the region state only involves a full-time query, and no further research has been done on the query by period. And it does not consider issues such as physical storage and dynamic structure adjustment of blockchain and partitioned consensus. These are interesting research directions toward realizing physical multi-chains suitable for IoV. 

## Figures and Tables

**Table 1:** Comparison between our scheme and the technical schemes related to geolocation and blockchain.

Literature	Storage Location	Coding Scheme	Indexes Mode	Function
[7]	smart contract	longitude and latitude	-	cooperative positioning
[8]	smart contract	hops between vehicles	-	credibility
[36]	smart contract	longitude and latitude	horizontal inspection and vertical inspection	privacy location queries
[9]	smart contract	longitude and latitude	-	improve the consensus efficiency
[17]	transaction	longitude and latitude	-	reliability verification
our scheme	transaction	Geohash	region state trie(RST)	regional information query

## Data Availability

Not applicable.
